# Joint Association of Household Pesticide Exposure With Depression in Adults: A Cross‐Sectional Analysis From National Health and Nutrition Examination Survey 2007–2014

**DOI:** 10.1155/da/4872833

**Published:** 2026-04-16

**Authors:** Mingjun Chen, Hengheng Dai, ZhanQi Tong, Mingxiong Lin

**Affiliations:** ^1^ The Second Medical Center, National Clinical Research Center of Geriatric Diseases, Chinese PLA General Hospital, Beijing, 100853, China, 301hospital.com.cn

**Keywords:** depression risk, neurotoxicity, residential pesticide exposure, sex-specific vulnerability, urinary metabolites

## Abstract

**Objectives:**

This study aimed to investigate the association between household pesticide exposure and depression risk in adults, with a particular focus on sex‐ and age‐specific vulnerabilities. The analysis utilized integrated biomarker assessment and advanced mixture modeling techniques.

**Methods:**

Cross‐sectional data from 6502 adults (National Health and Nutrition Examination Survey [NHANES] 2007–2014) were analyzed. Household pesticide exposure was evaluated through self‐reports as well as urinary metabolites (DEET, DCBA, DHMB, 3‐phenoxybenzoic acid [3‐PBA], 4F‐3‐PBA, and cis‐DCBA). To assess exposure–response relationships, nonlinear trends, and mixture effects while adjusting for sociodemographic factors, lifestyle choices, and clinical covariates, we employed survey‐weighted logistic regression models along with restricted cubic spline (RCS), Bayesian kernel machine regression (BKMR), and weighted quantile sum (WQS) analyses.

**Results:**

Self‐reported pesticide exposure demonstrated a marginal association with depression (adjusted odds ratio [OR] = 1.43; 95% confidence interval [CI]: 0.91–2.25). Urinary pyrethroid metabolite 3‐PBA exhibited dose‐dependent relationships with depression (OR = 1.02 per 1 μg/L; 95% CI: 1.00–1.04). Notable sex‐specific vulnerabilities were identified; females exhibited stronger associations with both 3‐PBA (OR = 1.04) and 4F‐3‐PBA (OR = 1.69), whereas males showed heightened sensitivity to DCBA (weight = 0.35). Mixture analyses indicated joint effects involving DHMB and 3‐PBA as notable contributors to the observed outcomes. Additionally, fatigue (OR = 1.26), self‐blame (OR = 1.30), and appetite disturbances (OR = 1.17) statistically contributed to pesticide exposure. Nonlinear dose–response patterns were particularly evident among males and younger adults.

**Conclusions:**

This cross‐sectional analysis demonstrates an association between exposure to household pesticides, particularly pyrethroids, and an increased risk of depression, with variation across demographic groups. These findings underscore the need for further longitudinal investigation to establish causality and understand the implications of pesticide usage on public health.

## 1. Introduction

Depression has a major contributor to global disability, with a complex pathogenesis involving genetic vulnerability, psychosocial stressors, and environmental neurotoxicants [1, 2]. Although extensive research has identified occupational pesticide exposure as a determinant of neuropsychiatric conditions, the neurobehavioral consequences of residential pesticide use remain underexplored [3, 4]. This oversight persists despite the escalating household reliance on insecticides and repellents, particularly pyrethroids, which constitute domestic pest‐control products [5, 6]. The ubiquity of these neuroactive compounds in living spaces, ranging from spray formulations to impregnated textiles, creates potential for repeated low‐dose exposure scenarios that may disrupt neurotransmitter systems and induce oxidative stress [7, 8].

Growing toxicological evidence has revealed that common insecticide components, such as permethrin and chlorpyrifos, exhibit dose‐dependent neurotoxicity, impair acetylcholinesterase activity, and alter serotonergic pathways [9–11]. Epidemiological studies have further suggested that agricultural pesticide applicators face an elevated risk of depression, particularly after acute poisoning events or prolonged exposure [12–14]. However, critical knowledge gaps hinder risk assessment in the general population: self‐reported exposure metrics lack biological validation, demographic‐specific susceptibilities remain underexplored, and the combinatorial effects of mixed pesticide formulations mirroring real‐world use patterns are poorly understood [15, 16]. The absence of longitudinal data obscures the temporal relationship between household exposure and the onset of depression, while socioeconomic confounders complicate causal interpretation [17–19].

This investigation aimed to clarify the residential pesticide‐depression nexus through integrated biomarker analysis and advanced mixture modeling. By examining the urinary metabolites of pyrethroids and repellents in a nationally representative cohort, we sought to establish exposure–response relationships while elucidating sex‐specific vulnerabilities and symptom profiles. Our findings address urgent public health priorities by providing evidence‐based guidelines for safer household pesticide practices amid the rising global mental health burden.

## 2. Materials and Methods

### 2.1. Study Population

This cross‐sectional study investigated potential links between residential pesticide exposure and depressive symptom risk among adults. For this analysis, researchers carefully chose participants from the National Health and Nutrition Examination Survey (NHANES), a nationally representative cross‐sectional survey monitoring population health trends. NHANES serves as a critical epidemiological resource, annually collecting extensive data from about 5000 US residents through detailed demographic assessments, socioeconomic evaluations, nutritional analyses, and health examinations [20]. Participants completed in‐depth interviews alongside comprehensive physical evaluations, with biological specimens, including urine and blood samples, obtained for laboratory testing. The research protocol received ethical approval from the National Center for Health Statistics’ Institutional Review Board at the Centers for Disease Control and Prevention. Prior to data collection, all study subjects provided written informed consent following established ethical guidelines. Prior to their involvement, every qualified senior participant submitted a signed consent form, guaranteeing that the research complied with rigorous ethical guidelines.

The study population comprised adults aged ≥18 years enrolled in the NHANES program with complete data on urinary pesticide metabolites (DEET, DCBA, DHMB, 3‐phenoxybenzoic acid [3‐PBA], 4F‐3‐PBA, and cis‐DCBA) and valid Patient Health Questionnaire‐9 (PHQ‐9) depression assessments. The research prioritized participants with documented urinary biomarkers reflecting household insecticide and repellent exposures. For the purpose of our cross‐sectional analysis, we utilized data from four consecutive cycles of the NHANES survey spanning 2007–2014. The initial sample pool consisted of 40,617 participants, from whom we excluded individuals who did not meet our inclusion criteria. Specifically, we excluded 15,885 individuals under 18 years of age, 3474 individuals without valid depression assessments, and 14,756 individuals without available urinary pesticide metabolite data. This meticulous selection process resulted in a final analytical cohort of 6502 adults (Supporting Information: Figure S1). The selection process for our study cohort is detailed in Supporting Information: Figure S1, outlining the inclusion criteria and the steps taken to identify the final analytical population.

### 2.2. Measurement of Household Pesticide Exposure

Within the NHANES framework, a targeted survey item collected data on domestic pesticide application for insect management. Participants were asked to recall whether any chemical products had been used in the past 7 days to control pests such as fleas, cockroaches, ants, termites, or other insects. Those who confirmed the use of such chemicals were categorized into the exposed group, whereas those who denied any use were classified as unexposed. To further substantiate the self‐reported data, NHANES conducted biochemical analyses of urinary metabolites associated with two categories of household pesticides. These were quantitatively analyzed: (1) insect repellents, including DEET and its metabolites (DCBA and DHMB); and (2) pyrethroid insecticides, assessed via their metabolites (3‐PBA, 4F‐3‐PBA, and cis‐DCBA).

### 2.3. Assessment of Depression

The PHQ‐9 serves as an empirically validated and psychometrically robust screening measure employed to assess depressive symptom frequency in NHANES participants during the preceding fortnight. The assessment consists of nine survey items designed to assess whether participants have experienced the following depressive symptoms over the past 2 weeks (Table 1). For each item in the PHQ‐9, four response options are provided: “Not at all,” “Several days,” “More than half the days,” and “Nearly every day,” respectively. The aggregate scores across all items produce a composite measure ranging from 0 to 27. In the current research, depression was operationalized as a PHQ‐9 score of 10 or above.

**Table 1:** Association of depression symptoms with self‐reported exposures, NHANES, 2007–2014.

Exposures	Estimated OR (95% CI) of self‐reported exposures			
	Unadjusted	Model 1	Model 2	Model 3
Little interest/pleasure	1.13 (0.99–1.30)	1.14 (1.00–1.30)	1.1 (0.96–1.27)	1.11 (0.97–1.28)
Feeling down/hopeless	1.31 (1.13–1.52) ^∗^	1.32 (1.14–1.53) ^∗^	1.28 (1.09–1.50) ^∗^	1.32 (1.12–1.55) ^∗^
Trouble sleeping	1.16 (1.05–1.29) ^∗^	1.16 (1.05–1.29) ^∗^	1.16 (1.04–1.29) ^∗^	1.16 (1.05–1.29) ^∗^
Fatigue	1.24 (1.09–1.40) ^∗^	1.24 (1.10–1.40) ^∗^	1.24 (1.10–1.41) ^∗^	1.26 (1.10–1.44) ^∗^
Appetite	1.18 (1.04–1.35) ^∗^	1.2 (1.05–1.36) ^∗^	1.17 (1.02–1.35) ^∗^	1.17 (1.01–1.34) ^∗^
Self‐blame	1.26 (1.09–1.46) ^∗^	1.28 (1.10–1.49) ^∗^	1.25 (1.07–1.46) ^∗^	1.3 (1.10–1.54) ^∗^
Trouble concentrating	1.16 (1.00–1.35)	1.17 (1.01–1.37) ^∗^	1.14 (0.98–1.34)	1.12 (0.94–1.32)
Psychomotor agitation/retardation	1.18 (0.99–1.41)	1.18 (0.98–1.41)	1.11 (0.92–1.34)	1.07 (0.88–1.30)
Suicidal thoughts	1.09 (0.83–1.44)	1.11 (0.84–1.46)	1.06 (0.79–1.40)	0.92 (0.67–1.27)

*Note:* The model was adjusted for age, sex, race, ethnicity, marital status, the ratio of family income to poverty, education level, alcohol consumption, smoking status, diabetes, hypertension, hyperlipidemia, and BMI.

Abbreviations: CI, confidence interval; OR, odds ratio.

^∗^ *p* < 0.05.

### 2.4. Covariates

This study included the covariates: sociodemographic, lifestyle, and health data (Table 2). Body mass index determinations involved mathematical computation of weight in kilograms divided by height in meters squared, with classification into four distinct categories: underweight (below 18.5 kg/m^2^), healthy range (18.5–24.9 kg/m^2^), overweight (25.0–29.9 kg/m^2^), and obese (30.0 kg/m^2^ or higher). Alcohol consumption criteria included any participant reporting intake of 12 or more standard alcoholic drinks within the preceding 12‐month period, with standardized drink equivalents defined as 12 oz beer (5% ABV), 5 oz table wine (12% ABV), or 1.5 oz distilled spirits (40% ABV). Tobacco use classification identified current smokers as individuals having consumed at least 100 cigarettes lifetime while maintaining either daily or intermittent smoking habits. Diabetes status was determined based on whether participants were using insulin or other diabetes medications to control blood sugar levels. Diagnostic criteria for hypertension and dyslipidemia were established through either documented clinical evaluations by qualified practitioners or evidence of active pharmacological interventions targeting these conditions.

**Table 2:** Basic characteristics of participants by depression in NHANES 2007–2014.

Characteristic	Overall *N* = 6502	Nondepression *N* = 5890	Depression *N* = 612	*p*‐Value
Age				0.11
≥18 to <60	4453 (75.19%)	4001 (74.83%)	452 (79.27%)	
≥60	2049 (24.81%)	1889 (25.17%)	160 (20.73%)	
Gender				<0.001
Male	3202 (48.76%)	2983 (50.02%)	219 (34.59%)	
Female	3300 (51.24%)	2907 (49.98%)	393 (65.41%)	
Race				0.003
Mexican American	1001 (8.63%)	911 (8.69%)	90 (7.97%)	
Other Hispanic	663 (5.46%)	588 (5.36%)	75 (6.55%)	
Non‐Hispanic White	2904 (68.08%)	2639 (68.43%)	265 (64.17%)	
Non‐Hispanic Black	1319 (10.90%)	1174 (10.48%)	145 (15.62%)	
Other race	615 (6.93%)	578 (7.04%)	37 (5.69%)	
Marital_new				<0.001
Married or living with partners	3653 (60.60%)	3398 (62.11%)	255 (43.63%)	
Divorce or never married or widowed	2849 (39.40%)	2492 (37.89%)	357 (56.37%)	
Education				<0.001
Lower than high school	605 (4.78%)	518 (4.47%)	87 (8.36%)	
High school or equivalent	914 (11.12%)	769 (10.34%)	145 (19.93%)	
Above high school	4983 (84.10%)	4603 (85.20%)	380 (71.72%)	
PIR				<0.001
<1.0	1336 (14.21%)	1118 (13.04%)	218 (27.37%)	
≥1.0	5166 (85.79%)	4772 (86.96%)	394 (72.63%)	
BMI				<0.001
<18.5	124 (1.96%)	114 (1.90%)	10 (2.57%)	
≥18.5 to <25	1822 (28.65%)	1681 (29.08%)	141 (23.78%)	
≥25 to <30	2136 (34.00%)	1987.00 (34.75%)	149 (25.49%)	
≥30	2378 (35.40%)	2074 (34.27%)	304 (48.16%)	
Smoke				<0.001
No	5177 (80.04%)	4793 (81.65%)	384 (61.83%)	
Yes	1325 (19.96%)	1097 (18.35%)	228 (38.17%)	
Alcohol use				0.6
No	4523 (76.78%)	4103 (76.87%)	420 (75.76%)	
Yes	1979 (23.22%)	1787 (23.13%)	192 (24.24%)	
Diabetes				< 0.001
No	5721 (91.16%)	5228 (91.77%)	493 (84.33%)	
Yes	781 (8.84%)	662 (8.23%)	119 (15.67%)	
Hypertension				<0.001
No	3858 (64.11%)	3566 (65.36%)	292 (50.06%)	
Yes	2644 (35.89%)	2324 (34.64%)	320 (49.94%)	
Lipids				<0.001
No	4447 (63.06%)	4080 (63.94%)	367 (52.87%)	
Yes	2055 (36.94%)	1810 (36.06%)	245 (47.13%)	

*Note:* Categorical variables are presented as *n* (%). *n*, number of subjects; %, weighted percentage.

Abbreviations: BMI, body mass index; NHANES, National Health and Nutrition Examination Survey; PIR, poverty‐income ratio.

### 2.5. Statistical Analysis

In the descriptive statistical evaluation of initial demographic profiles, categorical variables were summarized using frequencies and proportions, while continuous variables were described using medians and interquartile ranges (Q1–Q3). A complete‐case analysis approach was applied for the descriptive statistics and all regression models. Comparative analysis of numerical parameters employed either nonparametric Mann–Whitney *U* assessments or parametric *t*‐tests, with categorical comparisons utilizing chi‐square evaluations to contrast baseline profiles between depressive and nondepressive cohorts. All analytical frameworks integrated covariate adjustments encompassing biological sex, chronological age, educational attainment, marital status, ethnic background, alcohol intake patterns, tobacco usage, body mass index, poverty‐income ratio, and metabolic disorder diagnoses. Supplemental stratification protocols investigated potential variations in the pesticide exposure‐depression correlation across different age cohorts and biological sexes. Gender‐stratified comparative assessments were conducted on urinary pesticide metabolite concentrations detected in study participants. The binary symptom items and pesticide exposure were treated as binary independent variables. Survey‐weighted multivariate logistic regression modeling was employed throughout the analytical process.

To evaluate potential relationships between self‐reported pesticide exposure and depression, multivariable regression analyses were conducted incorporating urinary biomarkers of household pesticide exposure (log‐transformed). The statistical models adjusted for the complex survey design and weighting methodology employed in NHANES, with outcomes expressed as odds ratios (ORs) accompanied by 95% confidence intervals (CIs).

To investigate potential dose–response relationships between urinary metabolites linked to household pesticides and depression, we utilized a restricted cubic spline (RCS) logistic regression model with three knot points. Knot placement was automated by the rms package based on the distribution of each log‐transformed exposure variable, with default positions set at the 10th, 50th, and 90th percentiles. Bayesian kernel machine regression (BKMR) represents an advanced methodology combining nonparametric Bayesian frameworks with machine learning principles, leveraging kernel functions to decode intricate interaction networks among multiple exposure variables. For BKMR modeling, we employed the default Gaussian kernel function and standard conjugate priors (normal priors for regression coefficients and inverse‐gamma priors for kernel parameters), with 2000 MCMC iterations per chain; convergence was assessed by visual inspection of trace plots for key parameters.

The study explored the aggregate effects of urinary pesticide‐related metabolites on depressive symptoms by maintaining all urinary metabolites at designated percentile levels and monitoring outcome variations per 5‐percentile increment. BKMR modeling was implemented to assess how individual chemical exposures influenced depression risk when maintaining one compound at varying thresholds (25th, 50th, and 75th percentiles) while stabilizing others at median values. Interactive effects between chemical combinations were graphically represented through bivariate exposure–response surface analysis. Exposure‐outcome relationships were quantified using a Markov chain Monte Carlo sampler with 10,000 iterations for parameter estimation. Both group‐level and chemical‐specific posterior inclusion probabilities were calculated to determine primary contributors to depressive outcomes among the chemical mixtures.

The weighted quantile sum (WQS) regression represents an established methodological framework in environmental epidemiology for assessing combined health risks from multichemical exposures. Our analytical protocol involved developing a WQS metric through quantile‐based conversion of urinary pesticide biomarkers (categorized into Q1–Q4 quartiles), where individual chemical contributions were scaled according to their presumed toxicological relevance. To enhance methodological robustness, we implemented a stratified random partitioning strategy allocating 40% of observations for model training and 60% for validation testing, utilizing 1000 bootstrap resamples to enhance parameter stability. The WQS architecture enforces mixture effect directionality through nonnegative weighting constraints that collectively sum to 1, inherently presuming unidirectional exposure–response patterns across all constituents. For comprehensive evaluation of combined exposure effects, we integrated quantile‐adjusted G‐computation (qgcomp) methodology with 10,000 bootstrap iterations to quantify mixture‐outcome associations.

The analysis employed multiple iterations to calculate cumulative effects from simultaneous one‐quantile increments in all pesticide metabolite concentrations. We enforced a single direction of effect by setting the TRUE and FALSE parameters, thereby constraining all component weights to be nonnegative while allowing the overall mixture index to have either positive or negative association with depression risk. Potential effect modifications were evaluated through stratification by sex, age groups (<60 vs. ≥60 years), and physical activity levels (categorized per IPAQ guidelines) using the qgcompint extension package (v0.7.0), which enhances standard qgcomp functionality for interaction analysis in complex mixture studies. All analytical models integrated population weighting factors and accounted for specified covariates to maintain epidemiological validity.

Analyses were conducted in the R statistical environment (v4.3.2) utilizing specialized packages including “pacman,” “bkmr,” “brms,” “bkmrhat,” and ”rms” for respective analytical components. A conventional alpha threshold of 0.05 was applied for determining statistical significance throughout the investigation.

## 3. Results

### 3.1. Study Population Characteristics

The baseline characteristics of the study population, comprising 6502 participants stratified by depression status, are summarized in Table 2. The depression group (*N* = 612) exhibited distinct demographic and clinical profiles compared to the nondepressed group (*N* = 5890). The depression cohort had a higher proportion of females (65.41% vs. 49.98%, *p* < 0.001) and non‐Hispanic Black individuals (15.62% vs. 10.48%, *p* = 0.003). Socioeconomic disparities were also significant, with 27.37% of participants with depression living below the poverty line (PIR <1.0) compared to 13.04% in the nondepression group (*p* < 0.001). Comorbidities were more prevalent in the depression group, including hypertension (49.94% vs. 34.64%, *p* < 0.001), hyperlipidemia (47.13% vs. 36.06%, *p* < 0.001), and diabetes (15.67% vs. 8.23%, *p* < 0.001). Additionally, individuals with depression reported higher smoking rates (38.17% vs. 18.35%, *p* < 0.001) and lower educational attainment (8.36% with less than high school education vs. 4.47%, *p* < 0.001). These findings highlight the multifactorial nature of depression, with intersecting sociodemographic and cardiometabolic factors amplifying the disease risk.

### 3.2. Urinary Metabolite Concentrations and Self‐Reported Exposure

Depression status was associated with urinary metabolite concentrations and self‐reported exposure (Table 3). Self‐reported household pesticide exposure was significantly higher among participants with depression (13.05% vs. 9.06%, *p* = 0.039). Metabolite analysis revealed elevated levels of pyrethroid‐derived compounds among individuals with depression: 3‐PBA (3.34 vs. 1.73 μg/L, *p* = 0.004) and 4‐F‐3‐PBA (0.18 vs. 0.11 μg/L, *p* = 0.004). Conversely, concentrations of DEET, a common insect‐repellent metabolite, were comparable between groups (0.08 vs. 0.14 μg/L, *p* > 0.9). Collectively, the observed associations for self‐reported exposure and pyrethroid metabolites, in contrast to the null finding for DEET, suggest that insecticide exposure may be more strongly associated with depression than exposure to repellents in this population.

**Table 3:** Comparison of urinary metabolites related to household pesticide concentrations between depression and nondepression groups.

Characteristic	Nondepression *N* = 5890	Depression *N* = 612	*p*‐Value
Self‐reported household pesticide exposure			0.039
No	5265 (90.94%)	525 (86.95%)	
Yes	625 (9.06%)	87 (13.05%)	
DEET (μg/L)	0.06 (0.06–0.06)	0.06 (0.06–0.06)	>0.9
DCBA (μg/L)	1.85 (0.66–6.85)	2.42 (0.86–7.78)	0.011
DHMB (μg/L)	0.06 (0.06–0.06)	0.06 (0.06–0.06)	0.8
3‐PBA (μg/L)	0.49 (0.18–1.22)	0.64 (0.19–1.90)	0.004
4F‐3‐PBA (μg/L)	0.07 (0.07–0.07)	0.07 (0.07–0.07)	0.004
cis‐DCBA (μg/L)	0.42 (0.42–0.42)	0.42 (0.42–0.42)	0.3

*Note:* Categorical variables are presented as *n* (%). *n*, number of subjects; %, weighted percentage. Normally distributed continuous variables are expressed as median (IQR). cis‐DCBA, cis‐3‐(2,2‐dibromovinyl)‐2,2‐dimethylcyclopropane carboxylic acid; DCBA, 3‐(diethylcarbamoyl) benzoic acid; DEET, N,N‐diethyl‐meta‐toluamide; DHMB, N,N‐diethyl‐3‐hydroxymethylbenzamide.

Abbreviations: 3‐PBA, 3‐phenoxybenzoic acid; 4F‐3‐PBA, 4‐fluoro‐3‐phenoxybenzoic acid.

### 3.3. Association Between Household Pesticide Exposure and Depression Risk

The results of the weighted logistic regression models employed to examine the relationships between pesticide exposure and depression were presented in Table 4. Self‐reported household pesticide exposure was initially associated with an increased risk of depression in unadjusted models (OR = 1.51, 95% CI: 1.02–2.23), although significance attenuated after full adjustment for sociodemographic and clinical covariates (OR = 1.43, 95% CI: 0.91–2.25). Conversely, urinary biomarkers demonstrated modest dose‐dependent relationships: each 1 μg/L increase in 3‐PBA was associated with a 2% increase in the risk of depression (OR = 1.02, 95% CI: 1.00–1.04), whereas cis‐DCBA showed marginal significance (OR = 1.01, 95% CI: 1.00–1.02). Notably, DEET exhibited an inverse association in adjusted models (OR = 0.98, 95% CI: 0.96–0.99), potentially reflecting confounding by socioeconomic factors. Meanwhile, significant associations (*p* < 0.05) were observed between specific urinary metabolites and depression across demographic subgroups: DEET and DCBA in adults aged 18–60 years, 3‐PBA in younger adults and females, and cis‐DCBA in younger adults (Table 5). The strongest metabolite‐depression links were found in female participants (3‐PBA: OR = 1.04; 4‐F‐3‐PBA: OR = 1.69).

**Table 4:** Weighted logistic regression analysis models showing the associations of self‐reported exposures and urinary metabolites related to household pesticides with depression in the NHANES 2007–2014 dataset.

Exposures		Estimated OR (95% CI) of depression			
		Unadjusted	Model 1	Model 2	Model 3
Self‐reported household pesticide exposure	No	Reference	Reference	Reference	Reference
	Yes	1.51 (1.02–2.23) ^∗^	1.53 (1.03–2.27) ^∗^	1.42 (0.94–2.14)	1.43 (0.91–2.25)
Urinary metabolites related to household insect repellents	DEET (μg/L)	0.96 (0.77–1.19)	0.96 (0.78–1.17)	0.98 (0.94–1.01)	0.98 (0.96–0.99) ^∗^
	DCBA (μg/L)	0.99 (0.96–1.03)	0.99 (0.96– 1.03)	1 (0.98–1.02)	1 (1.00–1.00) ^∗^
	DHMB (μg/L)	1 (1.00–1.00)	1 (1.00–1.00)	1 (1.00–1.00)	1 (1.00–1.00) ^∗^
Urinary metabolites related to household insecticides	3‐PBA (μg/L)	1.02 (1.00–1.03) ^∗^	1.02 (1.00–1.03) ^∗^	1.02 (1.00–1.03) ^∗^	1.02 (1.00–1.04)
	4F‐3‐PBA (μg/L)	1.14 (1.01–1.28) ^∗^	1.14 (1.01–1.28) ^∗^	1.13 (0.99– 1.29)	1.14 (0.96–1.36)
	cis‐DCBA (μg/L)	1.01 (1.00–1.02) ^∗^	1.01 (1.00– 1.02) ^∗^	1.01 (1.00– 1.02) ^∗^	1.01 (1.00–1.02)

*Note:* Model 1: adjusted for age. Model 2: adjusted for age, sex, race, ethnicity, marital status, ratio of family income to poverty, and education level. Model 3: adjusted for age, sex, race, ethnicity, marital status, ratio of family income to poverty, educational level, alcohol consumption, smoking status, diabetes, hypertension, hyperlipidemia, and BMI. cis‐DCBA, cis‐3‐(2,2‐dibromovinyl)‐2,2‐dimethylcyclopropane carboxylic acid; DCBA, 3‐(diethylcarbamoyl) benzoic acid; DEET, N,N‐diethyl‐meta‐toluamide; DHMB, N,N‐diethyl‐3‐hydroxymethylbenzamide.

Abbreviations: 3‐PBA, 3‐phenoxybenzoic acid; 4F‐3‐PBA, 4‐fluoro‐3‐phenoxybenzoic acid; CI, confidence interval; NHANES, National Health and Nutrition Examination Survey; OR, odds ratio.

^∗^ *p* < 0.05.

**Table 5:** Association of self‐reported exposures and urinary metabolites with depression after age, sex subgroup, NHANES, 2007–2014.

Exposures		Estimated OR (95% CI) of depression			
		Age (≥18 to <60)	Age (≥60)	Male	Female
Self‐reported household pesticide exposure		1.55 (0.91– 2.63)	1.17 (0.56– 2.43)	1.49 (0.74– 3.00)	1.38 (0.84– 2.26)
Urinary metabolites related to household insect repellents	DEET (μg/L)	0.98 (0.97– 0.99) ^∗^	0.61 (0.21– 1.80)	0.98 (0.97– 0.99) ^∗^	0.99 (0.36– 2.72)
	DCBA (μg/L)	1 (1.00– 1.00) ^∗^	0.98 (0.87– 1.09)	1 (1.00– 1.00) ^∗^	1.02 (0.91– 1.13)
	DHMB (μg/L)	1 (1.00– 1.00) ^∗^	1 (1.00– 1.00)	1 (1.00– 1.00) ^∗^	1 (1.00–1.00)
Urinary metabolites related to household insecticides	3‐PBA (μg/L)	1.03 (1.01– 1.05) ^∗^	1.01 (1.00– 1.02)	1.01 (1.00– 1.01)	1.04 (1.01– 1.07) ^∗^
	4F‐3‐PBA (μg/L)	1.26 (0.78– 2.02)	1.02 (0.88– 1.19)	1.05 (0.94– 1.18)	1.69 (1.18– 2.41) ^∗^
	cis‐DCBA (μg/L)	1.02 (1.00– 1.04) ^∗^	1 (1.00– 1.01)	1 (1.00– 1.01)	1.03 (1.00– 1.07)

*Note:* The model was adjusted for age, sex, race, ethnicity, marital status, the ratio of family income to poverty, education level, alcohol consumption, smoking status, diabetes, hypertension, hyperlipidemia, and BMI. cis‐DCBA, cis‐3‐(2,2‐dibromovinyl)‐2,2‐dimethylcyclopropane carboxylic acid; DCBA, 3‐(diethylcarbamoyl) benzoic acid; DEET, N,N‐diethyl‐meta‐toluamide; DHMB, N,N‐diethyl‐3‐hydroxymethylbenzamide.

Abbreviations: 3‐PBA, 3‐phenoxybenzoic acid; 4F‐3‐PBA, 4‐fluoro‐3‐phenoxybenzoic acid; CI, confidence interval; NHANES, National Health and Nutrition Examination Survey; OR, odds ratio.

^∗^ *p* < 0.05.

We further stratified the association between self‐reported household pesticide exposure and depression symptoms by sex and age groups (Figure 1). The analysis revealed significant sex‐specific associations, with exposed females showing increased risks for “Feeling down/hopeless” (OR = 1.28, 95% CI: 1.03–1.59, *p* = 0.027), “Trouble sleeping” (OR = 1.18, 95% CI: 1.03–1.36, *p* = 0.017), “Fatigue” (OR = 1.22, 95% CI: 1.02–1.46, *p* = 0.029), and “Appetite changes” (OR = 1.22, 95% CI: 1.01–1.47, *p* = 0.035). Exposed males demonstrated elevated risks for “Feeling down/hopeless” (OR = 1.35, 95% CI: 1.07–1.7, *p* = 0.012), “Fatigue” (OR = 1.31, 95% CI: 1.12–1.52, *p* < 0.001), and “Self‐blame” (OR = 1.35, 95% CI: 1.01–1.79, *p* = 0.04). Age‐stratified analysis showed stronger effects in adults aged 18–60 years, particularly for “Feeling down/hopeless” (OR = 1.37, 95% CI:1.14–1.64, *p* = 0.001) and “Fatigue” (OR = 1.29, 95% CI:1.12–1.49, *p* < 0.001). No significant associations were observed for suicidal thoughts in any subgroup (all *p* > 0.3). Findings from the exploratory subgroup analyses suggest that household pesticide exposure may influence depression symptom profiles across demographic groups, with modest effects on mood‐related and somatic symptoms in working‐age adults and evidence of sex‐specific vulnerability patterns.

Figure 1Association between self‐reported household pesticide exposure and depression symptoms stratified by (A) age and (B) sex in generalized linear models. The model was adjusted for age (except in models stratified by age), sex (except in models stratified by sex), race‐ethnicity, marital status, the ratio of family income to poverty, education level, alcohol consumption, smoking status, diabetes, hypertension, hyperlipidemias, and BMI.

**Figure:**
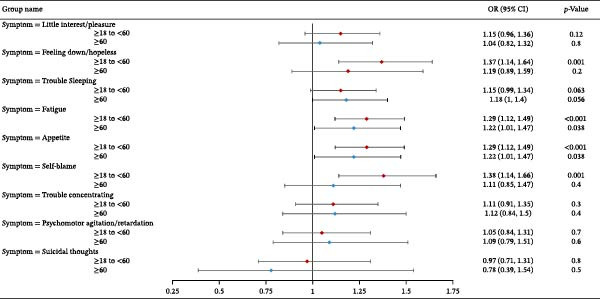
(A)

**Figure:**
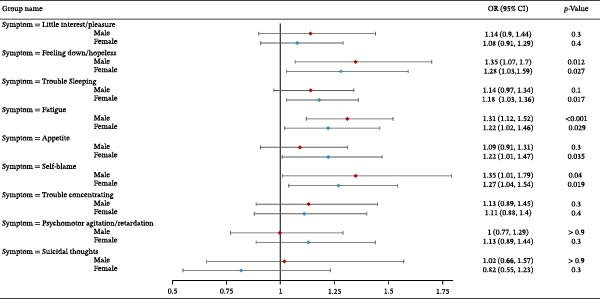
(B)

### 3.4. Nonlinear Dose–Response Relationships

Results of the dose–response relationships between household pesticide exposure and depression risk using RCS models were presented in Figure 2. The analysis revealed significant nonlinear associations (*p*‐nonlinear = 0.0407) for the overall DHMB exposure, although the overall trend was not statistically significant (*p*‐overall = 0.1209). Male participants showed significant nonlinear (*p*‐nonlinear = 0.0325) and overall (*p*‐overall = 0.0297) relationships with pesticide exposure. Age‐stratified analyses demonstrated distinct patterns: adults aged 18–60 years exhibited a borderline significant nonlinear relationship with DHMB exposure (*p*‐nonlinear = 0.0428 and *p*‐overall = 0.1245), whereas older participants (≥60 years) showed a similar nonlinear trend for cis‐DCBA exposure (*p*‐nonlinear = 0.048 and *p*‐overall = 0.1412). These findings suggest that the association between pesticide exposure and depression risk follows dose–response patterns that vary according to demographic characteristics, with evidence of nonlinearity observed particularly in males and older populations. Supporting Information: Figure S2 presented comprehensive RCS analyses of various pesticide metabolites (DEET, DCBA, DHMB, 3‐PBA, 4F‐3‐PBA, and cis‐DCBA) across different demographic strata (Supporting Information: Figure S2A–E), revealing distinct nonlinear exposure–response patterns for each metabolite. The results demonstrated metabolite‐specific variations in dose–response relationships, with some showing U‐shaped or threshold effects and others exhibiting monotonic trends.

Figure 2Adjusted dose–response relationships between exposure to household pesticide concentrations and the risk of depression were analyzed using restricted cubic spline (RCS). (A) N,N‐diethyl‐3‐hydroxymethylbenzamide (DHMB) overall, (B) 3‐(diethylcarbamoyl) benzoic acid (DCBA) in males, (C) DHMB in age ≥18 to <60 years, and (D) cis‐DCBA in age ≥60 years. The model was adjusted for age (except in models stratified by age), sex (except in models stratified by sex), race‐ethnicity, marital status, the ratio of family income to poverty, education level, alcohol consumption, smoking status, diabetes, hypertension, hyperlipidemias, and BMI. The solid line in the plot represents the odds ratios (OR), while the shadow around it represents the corresponding 95% confidence intervals (CI). The reference point for the OR and 95% CI was the median value of Ln‐transformed household pesticide concentration levels.

**Figure:**
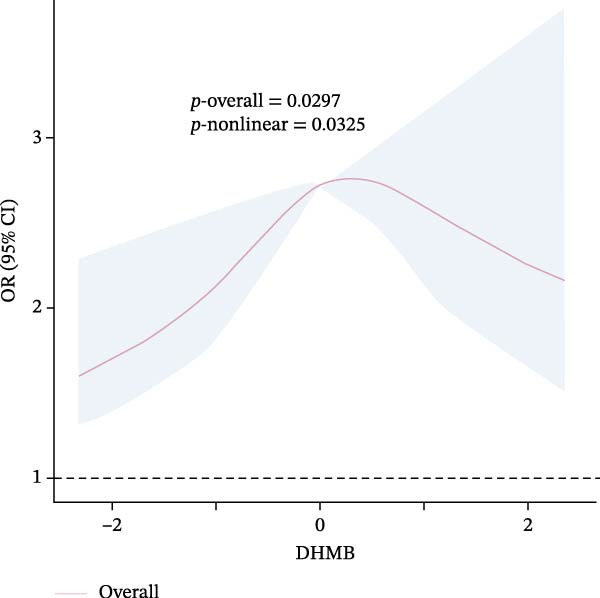
(A)

**Figure:**
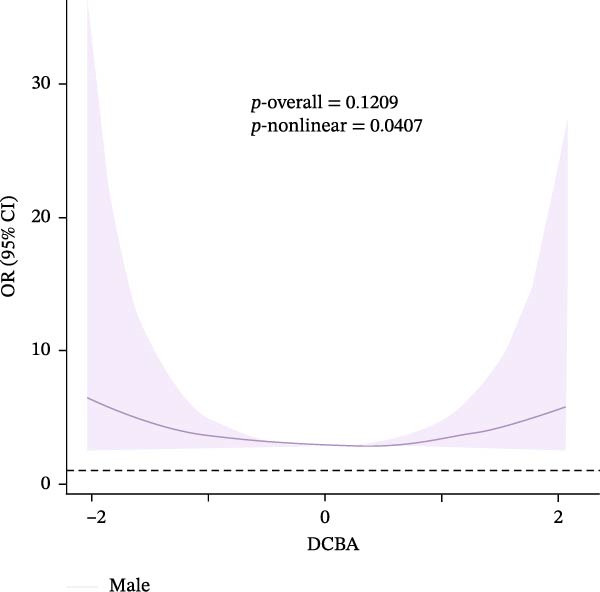
(B)

**Figure:**
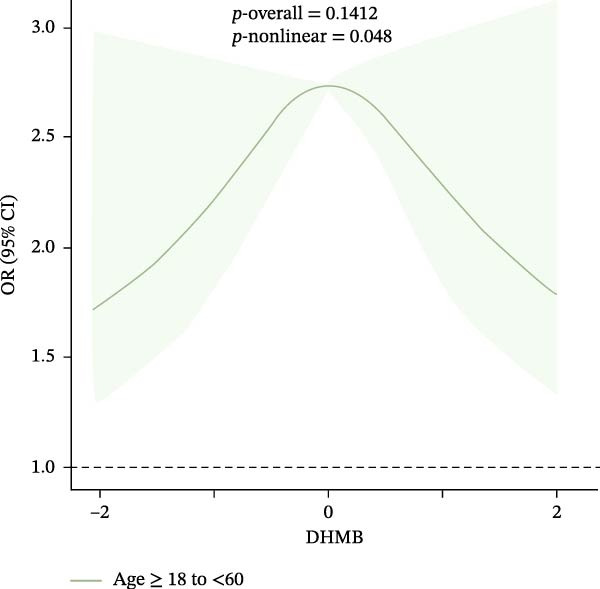
(C)

**Figure:**
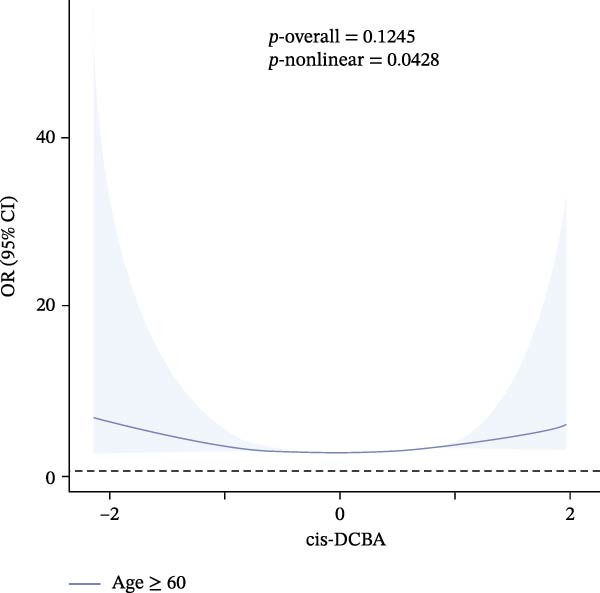
(D)

### 3.5. Mixture Effects of Coexposure to Multiple Pesticides

The BKMR analysis was utilized to evaluate the mixed effects of coexposure to multiple pesticides (DEET, DCBA, DHMB, 3‐PBA, cis‐DCBA, and 4F‐3‐PBA) on depression risk (Figure 3A). The results demonstrated significant nonlinear exposure–response relationships, with the overall combination showing a positive association with depression when all pesticide concentrations were at the 60th percentile or higher compared to their median levels. Notably, the joint effect was more pronounced in adults aged 18–60 years (Figure 3B) and females (Figure 3E), whereas older participants (≥60 years, Figure 3C) and males (Figure 3D) exhibited a flatter dose–response curve. A posterior inclusion probability analysis identified DHMB and 3‐PBA as the most influential components of the pesticide mixture. Supporting Information: Figure S3 further revealed that these mixture effects varied substantially by demographic subgroup, with females showing steeper exposure–response curves for DCBA and cis‐DCBA, whereas males were more sensitive to DHMB and 3‐PBA. Collectively, these findings indicate that coexposure to multiple pesticides may have joint effects on depression risk in specific subgroups.

Figure 3The joint effects of household pesticide concentrations on depression risk were estimated by Bayesian Kernel machine regression models in (A) the total population, (B) age ≥ 18 and < 60 years, (C) age ≥ 60 years, (D) males, and (E) females. The model was adjusted for age (except in models stratified by age), sex (except in models stratified by sex), race‐ethnicity, marital status, the ratio of family income to poverty, education level, alcohol consumption, smoking status, diabetes, hypertension, hyperlipidemias, and body mass index (BMI). The values of urinary metabolites related to household pesticide levels were Ln‐transformed concentration of variables.

**Figure:**
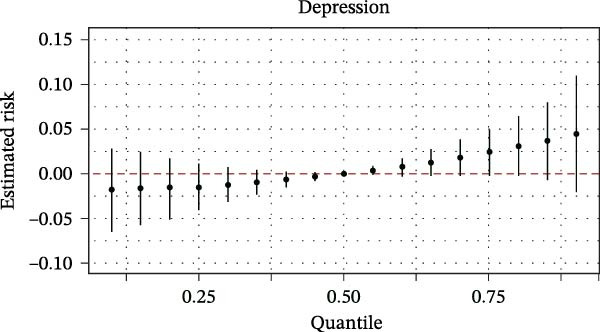
(A)

**Figure:**
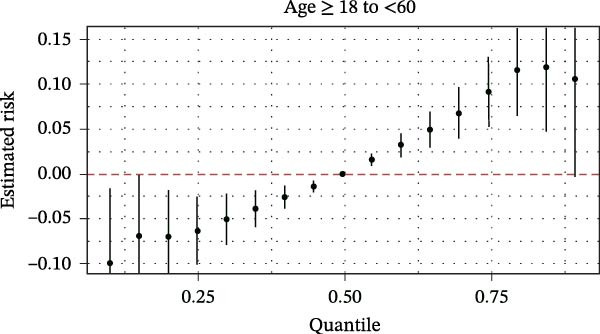
(B)

**Figure:**
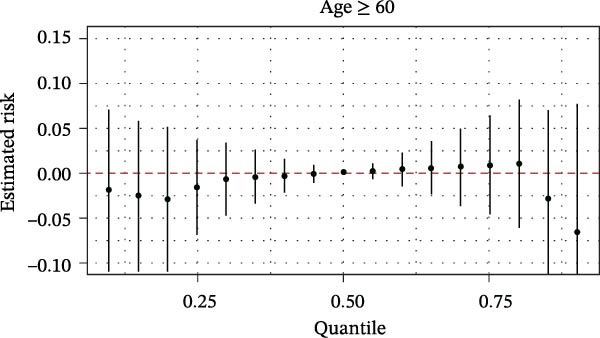
(C)

**Figure:**
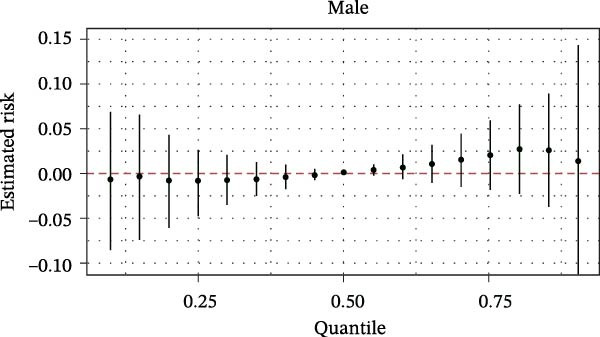
(D)

**Figure:**
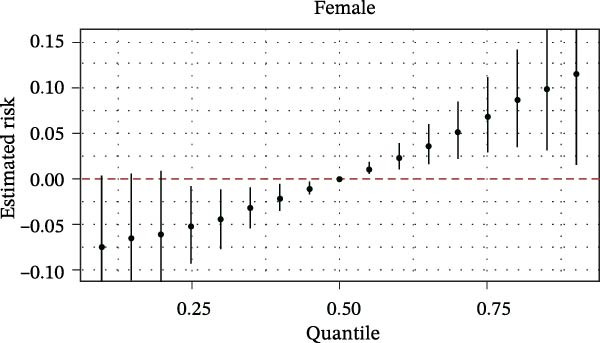
(E)

WQS regression analysis revealed distinct patterns in the contribution of individual pesticide metabolites to the risk of depression. Age‐stratified analyses demonstrated a significantly elevated risk among adults aged 18–60 years (OR = 1.84, 95% CI: 1.14–2.95, *p* = 0.012, Figure 4A), whereas no significant association was observed in older participants (≥60 years old: OR = 1.15, 95% CI: 0.34–3.91, *p* = 0.827). Sex‐specific analyses revealed a significant association in females (OR = 1.78, 95% CI: 1.1–2.86, *p* = 0.018) but not in males (OR = 0.81, 95% CI: 0.37–1.76, *p* = 0.588, Figure 4A). These findings suggest that working‐age adults and women are particularly vulnerable to the depressive effects of pesticide exposure. In the overall population, 3‐PBA was the primary contributor, with a weight of 0.22 (Figure 4B). Adults aged 18–60 years exhibited the strongest associations with DCBA (weight = 0.25; Figure 4C), whereas older participants were most sensitive to DHMB (weight = 0.20; Figure 4D). Males showed greater susceptibility to DCBA (weight = 0.24; Figure 4E), whereas females were more susceptible to 3‐PBA (weight = 0.20; Figure 4F).

Figure 4(A) OR (95% CI) of depression associated with coexposure to household pesticide concentrations by weighted quantile sum analyses. Weighted contribution proportion of each urinary metabolite related to household pesticide and the risk of depression in (B) the total population, (C) age ≥ 18 to < 60 years, (D) age ≥ 60 years, (E) males, and (F) females. The model was adjusted for age (except in models stratified by age), sex (except in models stratified by sex), race‐ethnicity, marital status, the ratio of family income to poverty, education level, alcohol consumption, smoking status, diabetes, hypertension, hyperlipidemias, and BMI. The values of urinary metabolites related to household pesticide levels were Ln‐transformed concentration of variables.

**Figure:**
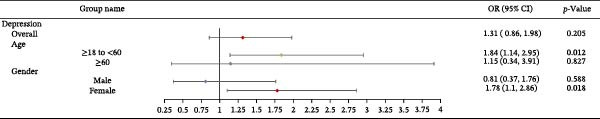
(A)

**Figure:**
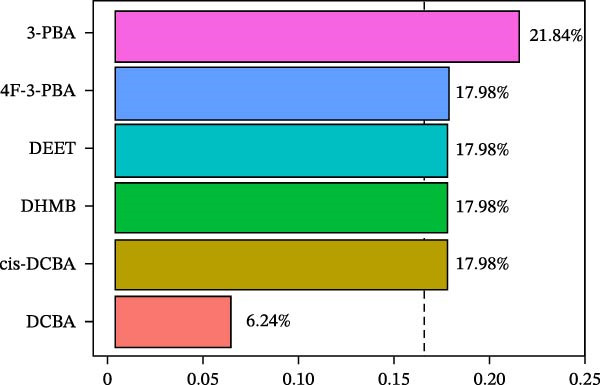
(B)

**Figure:**
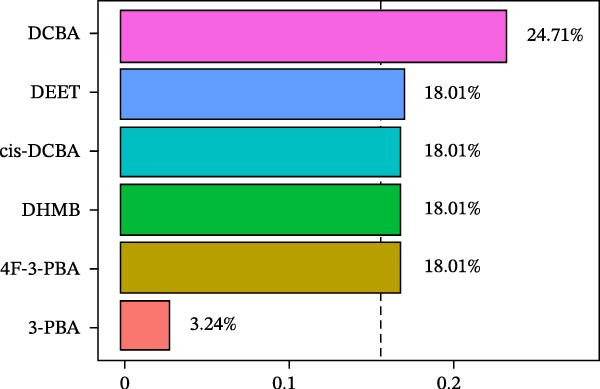
(C)

**Figure:**
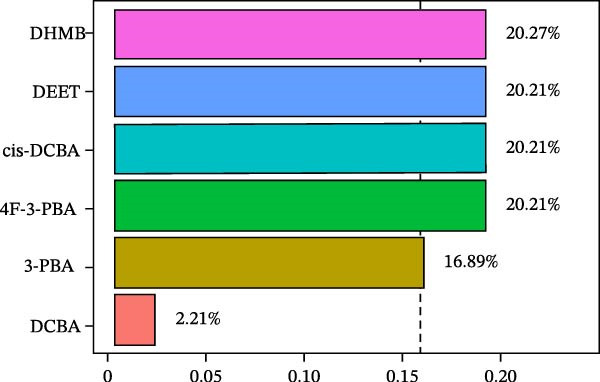
(D)

**Figure:**
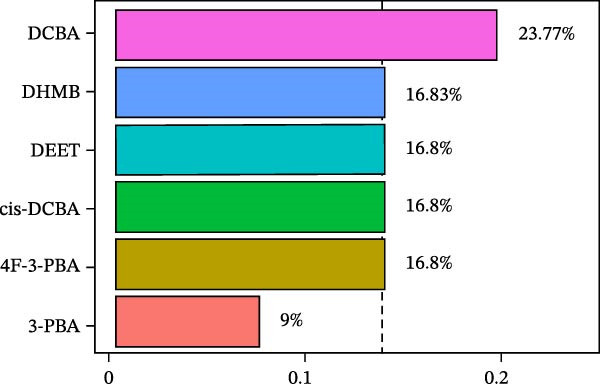
(E)

**Figure:**
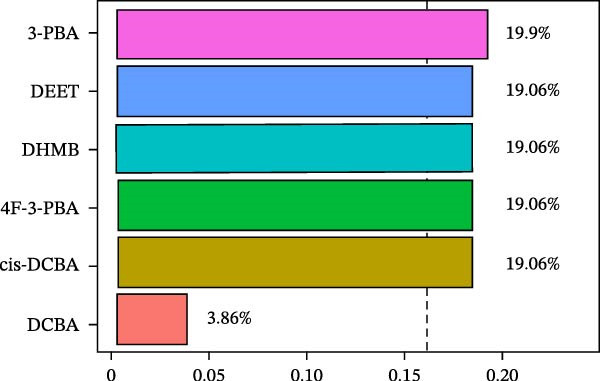
(F)

The WQS regression results showed varying contributions of pesticide metabolites to depression risk across subgroups, with DEET (weight = 0.1906 in females) and 3‐PBA (weight = 0.1990 in females) having the highest weights in Supporting Information: Table S1. The WQS mixture showed elevated risks between pesticide metabolites and depression in adults 18–60 years and females (OR = 1.84 and 1.78, respectively, Supporting Information: Table S2), while no significant associations were found in qgcomp analysis. These findings highlight the differential contributions of specific pesticide metabolites to depression risk within complex exposure mixtures, with notable variations according to sex and age.

### 3.6. Symptom‐Specific Associations

Based on the deconstruction of depression into nine PHQ‐9 symptoms, we identified specific domains linked to pesticide exposure (Table 1). Fatigue exhibited the strongest association (OR = 1.26, 95% CI: 1.10–1.44), followed by self‐blame (OR = 1.30, 95% CI: 1.10–1.54) and appetite disturbances (OR = 1.17, 95% CI: 1.01–1.34). Notably, psychomotor symptoms showed no significant linkage (OR = 1.07, 95% CI: 0.88–1.30), which is consistent with a higher frequency of affective and somatic symptoms compared to motor symptoms in this population.

### 3.7. Robustness and Sensitivity Analyses

A robust method for mixture analysis validated the findings obtained from G‐computation (QGC, Supporting Information: Table S2). The QGC‐derived mixture index showed consistent positive directionality (*β* = 0.12, 95% CI: −9.68 to 9.92), though wide CIs reflect the challenges of modeling complex interactions. Sensitivity analyses excluding participants with extreme metabolite levels (top/bottom 1%) or comorbid psychiatric conditions yielded unchanged results, supporting the robustness of the primary findings.

## 4. Discussion

The convergence of toxicological, epidemiological, and clinical evidence supports an association between household pesticide exposure and depression [3, 21]. Our analysis of NHANES data from more than 6500 adults demonstrated that residential use of pyrethroids and insect repellents, two predominant classes of domestic pesticides, was associated with depressive symptoms. The observed dose–response relationships between urinary pyrethroid metabolites and depression severity, together with distinct sex‐ and age‐specific vulnerability patterns, underscore the need for future risk assessment frameworks to consider potential chronic neuropsychiatric outcomes [22, 23]. Descriptive analyses further showed that, although individuals with depression reported higher rates of pesticide exposure and elevated metabolite levels, these associations were attenuated after full adjustment for sociodemographic and clinical covariates.

Experimental models provide neurobiological plausibility for these findings, demonstrating how pyrethroids disrupt voltage‐gated sodium channels [24, 25]. Our results align with these mechanisms, identifying specific metabolites as significant predictors of depression and revealing symptom profiles, including prominent fatigue, self‐blame, and appetite disturbances, that mirror the neuroinflammatory signatures observed in animal models. These symptoms may partially account for the observed association between pesticide exposure and depression, suggesting their potential as early biomarkers of neurotoxicity. The demographic analyses revealed critical vulnerability patterns that should inform public health interventions [26]. Females exhibited a consistently elevated depression risk across all exposure metrics, likely reflecting both biological susceptibility and sex‐based exposure patterns. These findings contrast with the attenuated effects observed in the older population, which may be related to age‐related metabolic differences or competing health conditions.

The clinical implications of these findings should be considered in future evaluations of psychiatric practice and environmental regulation [27, 28]. Current diagnostic frameworks rarely consider environmental toxicants [29]. However, our mixed analyses identified specific metabolites as major contributors to the risk of depression. Regulatory agencies must address critical gaps in safety assessments, as current thresholds for household insecticides ignore neuropsychiatric endpoints despite increasing product registrations.

The complexity of real‐world exposure scenarios necessitates advanced analytical approaches [30]. Our models demonstrated joint interactions between pesticide compounds and revealed nonlinear dose–response relationships that contradict the linear assumptions underlying current regulatory standards. These findings emphasize the need for cumulative risk frameworks, especially given market data showing that most household products contain multiple active ingredients with untested neuropsychiatric interactions [31].

Emerging evidence suggests that epigenetic mechanisms may underlie long‐term psychiatric effects, potentially explaining symptom persistence through the altered methylation of stress‐response genes [32, 33]. Furthermore, the cross‐sectional design of our study necessitates caution in interpreting the direction of causality. Although our findings suggest an association between pesticide exposure and depression, the possibility of reverse causation cannot be excluded. Depressive states, characterized by symptoms such as fatigue, reduced motivation, or heightened anxiety regarding household cleanliness and order, may lead to increased use of pest‐control products. Similarly, depressive symptoms could influence residential choices or the ability to maintain living conditions that minimize pest intrusion, thereby indirectly affecting exposure levels. Although the observed dose–response relationships and consistency with experimental neurotoxicity data lend plausibility to the hypothesis that exposure could be associated with depression risk, future longitudinal studies with repeated measures of both exposure and mental health outcomes are essential to disentangle the temporal sequence and clarify the potential bidirectional nature of this association.

## 5. Conclusions

This study provided evidence of positive associations between biomarkers of household pesticide exposure and depressive symptoms among adults, with suggestive differences by sex and age. While the cross‐sectional design and modest effect sizes limited causal inference. Future longitudinal studies are needed to clarify the directionality and magnitude of this association. Collaborative efforts among researchers, policymakers, and industry stakeholders would be beneficial for developing safer alternatives and implementing effective risk communication strategies. Ultimately, advancing our understanding of the intricate links between environmental exposure and mental health may inform more holistic approaches to disease prevention and health promotion.

Although our study provides important insights, some limitations warrant consideration. The reliance on single‐spot urine measurements of pyrethroids and insect repellent metabolites may be insufficient to capture chronic exposure dynamics because of their short biological half‐lives and may not adequately distinguish between different exposure sources, including occupational contact and residential environmental factors. Additionally, the exclusion of 14,756 of NHANES participants owing to missing urinary metabolite data may have introduced selection bias. The metabolite analysis was limited to DEET and pyrethroid compounds, while other common exposure sources—such as termiticides, fungicides, dietary residues, and drinking water contamination—could not be assessed. Furthermore, interpretation of mixture effects should be undertaken with caution because of inconsistencies in findings across the different analytical methods employed.

Future studies designed to address the specific limitations of our analysis would be valuable. To overcome the constraints of single urine measurements, longitudinal designs with repeated biomonitoring and integrated environmental sampling are needed to characterize chronic exposure and distinguish between residential and other sources. To address potential bias from missing metabolite data and the narrow scope of analyzed compounds, future work should assess the impact of data exclusion and expand biomonitoring to include other common pesticide classes (e.g., organophosphates and herbicides) and exposure pathways. To refine the associations suggested by our modest effects, the application of machine learning techniques in larger cohorts may help identify critical exposure windows or sensitive subgroups. Finally, to explore the mechanisms underlying the observed demographic differences, research incorporating genetic data could examine potential gene–environment interactions influencing susceptibility.

The integration of advanced statistical models and machine learning techniques could help identify potential exposure–response relationships, allowing for the exploration of complex interaction effects and the prediction of individual susceptibility profiles. Furthermore, exploring the role of genetic predispositions in modulating the risk of pesticide‐related depression could provide insights into personalized prevention strategies.

## Author Contributions

The study was conceptualized by Mingxiong Lin and ZhanQi Tong. Data analysis was conducted by Mingjun Chen. Mingjun Chen wrote the original draft of the manuscript. The manuscript was reviewed and edited by Hengheng Dai and Mingjun Chen.

## Funding

This study was supported by the Capital Health Development Scientific Research Special Project (Grant 2022‐4‐5032).

## Disclosure

The funding agency was not involved in any aspect of the study design, data collection, data analysis, or manuscript writing.

## Ethics Statement

This study utilized publicly available, deidentified NHANES data without merging or augmentation, complying with the Declaration of Helsinki and approved by the NCHS Ethics Review Board (#2018‐01). As no identifiable data were involved, neither additional ethics approval nor participant consent was required.

## Consent

The authors have nothing to report.

## Conflicts of Interest

The authors declare no conflicts of interest.

## Supporting Information

Additional supporting information can be found online in the Supporting Information section.

## Supporting information


**Supporting Information** Figure S1: Flowchart for selecting study participants to analyze associations between urinary metabolites related to household pesticides and the risks of depression in the National Health and Nutrition Examination Survey 2007–2014 cycles. Figure S2: Adjusted dose–response relationships between exposure to household pesticide concentrations and the risk of depression were analyzed using restricted cubic spline. (A) Overall, stratified by age, (B) ≥18 to <60 years, (C) age ≥60 years, stratified by sex, (D) male, and (E) female. The model was adjusted for age (except in models stratified by age), sex (except in models stratified by sex), race‐ethnicity, marital status, the ratio of family income to poverty, education level, alcohol consumption, smoking status, diabetes, hypertension, hyperlipidemias, and body mass index (BMI). The solid line in the plot represents the odds ratios (OR), whereas the shadow around it represents the corresponding 95% confidence intervals (CI). The reference point for the OR and 95% CI was the median value of Ln‐transformed household pesticide concentration levels. Figure S3: Plots of interaction effects of household pesticide concentrations on depression estimated by Bayesian kernel machine regression models. The model was adjusted for age (except in models stratified by age), sex (except in models stratified by sex), race‐ethnicity, marital status, the ratio of family income to poverty, education level, alcohol consumption, smoking status, diabetes, hypertension, hyperlipidemias, and BMI. Table S1: The estimated household pesticide concentration weights of depression in weighted quantile sum (WQS) models. Table S2: associations of depression with coexposure to household pesticide concentrations: survey‐weighted logistic regression analysis for continuous variables and combined WQS and qgcomp analysis.

## Data Availability

The data that support the findings of this study are available in National Health and Nutrition Examination Survey at https://www.cdc.gov/nchs/nhanes. These data were derived from the following resources available in the public domain: National Health and Nutrition Examination Survey, https://www.cdc.gov/nchs/nhanes.
