# Supplementary material for: Joint Association of Household Pesticide Exposure With Depression in Adults: A Cross‐Sectional Analysis From National Health and Nutrition Examination Survey 2007–2014
Source: Depress Anxiety. 2026 Apr 16;2026:4872833. doi: 10.1155/da/4872833 (PMC13087505; doi:10.1155/da/4872833)
Supplement: Supplementary file 1 — Supporting Information Figure S1: Flowchart for selecting study participants to analyze associations between urinary metabolites related to household pesticides and the risks of depression in the National Health and Nutrition Examination Survey 2007–2014 cycles. Figure S2: Adjusted dose–response relationships between exposure to household pesticide concentrations and the risk of depression were analyzed using restricted cubic spline. (A) Overall, stratified by age, (B) ≥18 to <60 years, (C) age ≥60 years, stratified by sex, (D) male, and (E) female. The model was adjusted for age (except in models stratified by age), sex (except in models stratified by sex), race‐ethnicity, marital status, the ratio of family income to poverty, education level, alcohol consumption, smoking status, diabetes, hypertension, hyperlipidemias, and body mass index (BMI). The solid line in the plot represents the odds ratios (OR), whereas the shadow around it represents the corresponding 95% confidence intervals (CI). The reference point for the OR and 95% CI was the median value of Ln‐transformed household pesticide concentration levels. Figure S3: Plots of interaction effects of household pesticide concentrations on depression estimated by Bayesian kernel machine regression models. The model was adjusted for age (except in models stratified by age), sex (except in models stratified by sex), race‐ethnicity, marital status, the ratio of family income to poverty, education level, alcohol consumption, smoking status, diabetes, hypertension, hyperlipidemias, and BMI. Table S1: The estimated household pesticide concentration weights of depression in weighted quantile sum (WQS) models. Table S2: associations of depression with coexposure to household pesticide concentrations: survey‐weighted logistic regression analysis for continuous variables and combined WQS and qgcomp analysis. [file DA-2026-4872833-s001.docx]

**Joint association of household pesticide exposure with depression in adults: A cross-sectional analysis from National Health and Nutrition Examination Survey 2007–2014**

Mingjun Chen^1*^, Hengheng Dai^1*^, ZhanQi Tong^1^, Mingxiong Lin^1#^

^1^ The Second Medical Center & National Clinical Research Center of Geriatric Diseases, Chinese PLA General Hospital, Beijing, 100853, China.

**Corresponding author:**

[13661175460@163.com](mailto:13661175460@163.com) (Mingxiong Lin).

This study was founded by the Capital Health Development Scientific Research Special Project (2022-4-5032).

**Figure S1. The flow charts of selecting study participants in analyzing of the associations of urinary metabolites related to household pesticide with risks of depression in the NHANES 2007-2014 cycles.**

A total of 40,617 individuals were included in NHANES 2007-2014

A total of 24,732 adult individuals were included

Excluded 15,885 individuals < 18 years old.

A total of 21,258 adult individuals with PHQ-9 scores data

Excluded 3,474 individuals without the PHQ-9 scores data

A total of 6,502 adult individuals remained

Excluded 14,756 individuals without the data on urinary DEETs

0 ≤ PHQ-9 score ≤ 9

Control Group （N = 5,890）

10 ≤ PHQ-9 score ≤ 27

Depression Group （N = 612）

**Figure S2. Adjusted dose–response relationships between exposure to household pesticide concentrations and the risk of depression was analyzed using restricted cubic spline (RCS). (A) overall, stratified by age (B) ≥ 18 to < 60, (C) age ≥ 60, stratified by sex (D) male, and (E) female. Model was adjusted for age (except in models stratified by age), sex (except in models stratified by sex), race-ethnicity, marital status, ratio of family income to poverty, education level, alcohol consumption, smoking status, diabetes, hypertension, hyperlipidemias, and BMI. The solid line in the plot represents the odds ratios (OR), while the shadow around it represents the corresponding 95 % confidence intervals (CI). The reference point for the OR and 95 % CI was the median value of Ln-transformed household pesticide concentrations levels.**


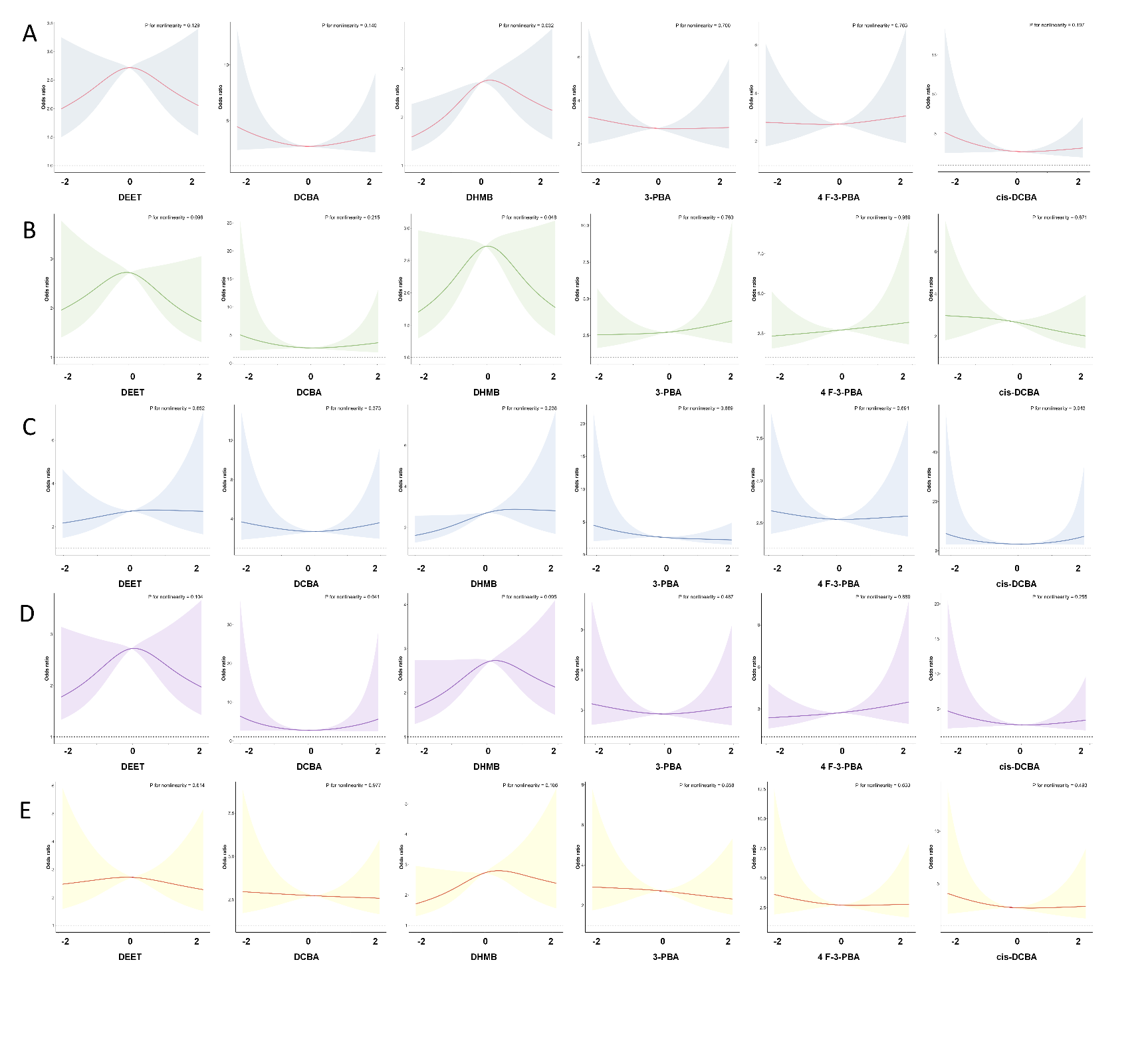


**Figure S3. Plots of interaction effects of household pesticide concentrations on depression estimated by BKMR models. Model was adjusted for age (except in models stratified by age), sex (except in models stratified by sex), race-ethnicity, marital status, ratio of family income to poverty, education level, alcohol consumption, smoking status, diabetes, hypertension, hyperlipidemias, and BMI.**

**
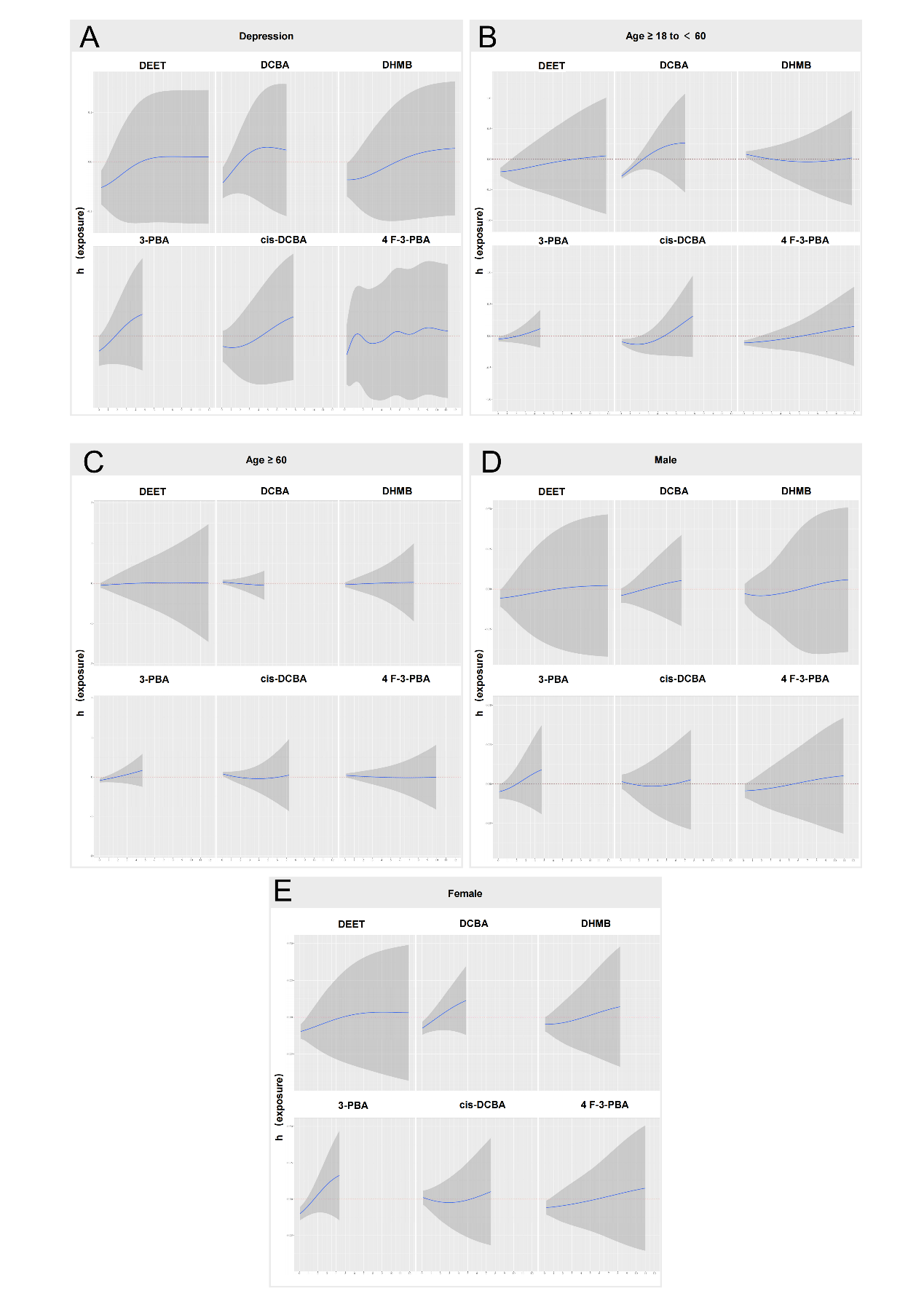
**

**Table S1. The estimated household pesticide concentrations weights of depression in WQS models.**

| **Variables** | **Mean-weight** | **Variables** | **Mean-weight** | **Variables** | **Mean-weight** |
| --- | --- | --- | --- | --- | --- |
| **Overall** |  | **Age (18-59)** |  | **Age (≥60)** |  |
| DEET | 0.1798 | DEET | 0.1801 | DEET | 0.2021 |
| DCBA | 0.0624 | DCBA | 0.2471 | DCBA | 0.0221 |
| DHMB | 0.1798 | DHMB | 0.1801 | DHMB | 0.2027 |
| 3-PBA | 0.2184 | 3-PBA | 0.0324 | 3-PBA | 0.1689 |
| 4 F-3-PBA | 0.1798 | 4 F-3-PBA | 0.1801 | 4 F-3-PBA | 0.2021 |
| **cis-DCBA** | **0.1798** | **cis-DCBA** | **0.1801** | **cis-DCBA** | **0.2021** |
|  |  | **Male** |  | **Female** |  |
|  |  | DEET | 0.1680 | DEET | 0.1906 |
|  |  | DCBA | 0.2377 | DCBA | 0.0386 |
|  |  | DHMB | 0.1683 | DHMB | 0.1906 |
|  |  | 3-PBA | 0.0090 | 3-PBA | 0.1990 |
|  |  | 4 F-3-PBA | 0.1680 | 4 F-3-PBA | 0.1906 |
|  |  | cis-DCBA | 0.1680 | cis-DCBA | 0.1906 |

The values of urinary metabolites related to household pesticide levels were ln-transformed concentration of variables.

**Table S2. Associations of depression with co-exposure to household pesticide concentrations: survey-weighted logistic regression analysis for continuous variables and mixture of WQS and qgcomp analysis.**

| **Variables** | **Overall** | **Age (≥ 18 to < 60)** | **Age (≥ 60)** | **Male** | **Female** |
| --- | --- | --- | --- | --- | --- |
|  | OR (95%CI) | OR (95%CI) | OR (95%CI) | OR (95%CI) | OR (95%CI) |
| **DEET, ug/L** | 0.98(0.96, 0.99) ^*^ | 0.98(0.97, 0.99) ^*^ | 0.61(0.21, 1.80) | 0.98(0.97, 0.99) ^*^ | 0.99(0.36, 2.72) |
| **DCBA, ug/L** | 1(1.00, 1.00) ^*^ | 1(1.00, 1.00) ^*^ | 0.98(0.87, 1.09) | 1(1.00, 1.00) ^*^ | 1.02(0.91, 1.13) |
| **DHMB, ug/L** | 1(1.00, 1.00) ^*^ | 1(1.00, 1.00) ^*^ | 1(1.00, 1.00) | 1(1.00, 1.00) ^*^ | 1(1.00, 1.00) |
| **3-PBA, ug/L** | 1.02(1.00, 1.04) | 1.03(1.01, 1.05) ^*^ | 1.01(1.00, 1.02) | 1.01(1.00, 1.01) | 1.04(1.01, 1.07) ^*^ |
| **4 F-3-PBA, ug/L** | 1.14(0.96, 1.36) | 1.26(0.78, 2.02) | 1.02(0.88, 1.19) | 1.05(0.94, 1.18) | 1.69(1.18, 2.41) ^*^ |
| **cis-DCBA, ug/L** | 1.01(1.00, 1.02) | 1.02(1.00, 1.04) ^*^ | 1(1.00, 1.01) | 1(1.00, 1.01) | 1.03(1.00, 1.07) |
| **Mixture of WQS** | 1.31 (0.86,1.98) | 1.84 (1.14,2.95) ^*^ | 1.15 (0.34,3.91) | 0.81 (0.37,1.76) | 1.78 (1.1,2.86) ^*^ |
| **Mixture of qgcomp** | 0.12 (-9.68,9.92) | 0.18 (-9.62,9.98) | -0.03 (-9.83,9.77) | 0.02 (-9.78,9.82) | 0.19 (-9.61,9.99) |

^*^ *P* < 0.05

Model was adjusted for age, sex, race-ethnicity, marital status, ratio of family income to poverty, education level, alcohol consumption, smoking status, diabetes, hypertension, hyperlipidemias, and BMI.
